# Testosterone influences renal electrolyte excretion in SHR/y and WKY males

**DOI:** 10.1186/1472-6793-8-5

**Published:** 2008-03-26

**Authors:** Jonathan Toot, Cathy Jenkins, Gail Dunphy, Shannon Boehme, Mike Hart, Amy Milsted, Monte Turner, Daniel Ely

**Affiliations:** 1 Biology Department, The University of Akron, Akron, OH 44325-3908, USA; 2 School of Biomedical Sciences, Kent State University, Kent, OH 44242, USA

## Abstract

**Background:**

The Y-chromosome (Yc) and testosterone (T) increase blood pressure and may also influence renal electrolyte excretion. Therefore, the goal of this study was to determine if the Yc combined with T manipulation could influence renal Na and K excretion.

**Methods:**

To investigate the role of the Yc and T, consomic borderline hypertensive (SHR/y) and normotensive Wistar-Kyoto (WKY) rat strains were used (15 weeks) in three T treatment groups: castrate, castrate with T implant and gonadally intact males. Urine was collected (24 hrs at 15 weeks of age) for Na and K measurements by flame photometry. RT-PCR was used to demonstrate the presence of renal androgen receptor (AR) transcripts. Plasma T and aldosterone were measured by RIA. In another experiment the androgen receptor was blocked using flutamide in the diet.

**Results:**

Na and K excretion were decreased by T in SHR/y and WKY. AR transcripts were identified in SHR/y and WKY kidneys. Plasma aldosterone was decreased in the presence of T. Blockade of the AR resulted in a significant increase in Na excretion but not in K excretion in both SHR/y and WKY males.

**Conclusion:**

T influences electrolyte excretion through an androgen receptor dependent mechanism. There was not a differential Yc involvement in electrolyte excretion between WKY and SHR/y males.

## Background

Human [1,2] and animal studies [3] have implicated Y-chromosome (Yc) loci as influencing the onset of male hypertension. In the Spontaneously Hypertensive Rat strain (SHR), the SHR Yc contains a locus which increases blood pressure (BP) approximately 20 mmHg compared to the normotensive Wistar Kyoto (WKY) Y chromosome. The Yc is only one genetic component of the total SHR hypertension. In SHR males, the presences of testosterone and androgen receptors through puberty are required for development of the hypertension and associated sympathetic nervous system potentiation, including the Yc component [4,5]. In examining phenotypes that mapped to the Yc and differed between SHR and WKY, our laboratory demonstrated an earlier rise of testosterone (T) levels in SHR males leading into puberty [4] and this phenotype mapped to the SHR Yc. Because of the unique biology of the mammalian Yc the blood pressure phenotype and the T timing phenotype cannot be separated using linkage. With the interaction of T and the development of the Yc hypertension, it is possible the two phenotypes are related and a single genetic locus is responsible for both phenotypes. In further support of a physiological T-SNS interaction [6], we have previously reported that T increases the storage and release of norepinephrine in isolated kidney studies more in SHR, as compared to the WKY males [7]. Reckelhoff *et al*. [8] reported that in SHR, T increased renal Na reabsorption, potentially through an androgen receptor (AR) mediated mechanism in the kidney. These results suggest a possible renal AR mediated response that may influence one of the renal BP control mechanisms.

The renal system operates under a well auto-regulated negative feedback system that maintains glomerular filtration rate and the filtered load of ions [9], as well as playing an important role in long-term BP regulation. As part of this regulation in genetic models of hypertension, the kidney requires a high level of arterial pressure to excrete Na and water [10] and the increase in renal arterial perfusion pressure results in increased excretion of water and Na. Fluctuations in BP and ion concentration can initiate renal and cardiovascular compensatory mechanisms through the renin angiotensin system, and aldosterone secretion [11]. Other studies comparing urinary Na excretion of SHR and WKY males [12,13] have found decreased urinary Na excretion and a reduced renal ability to excrete Na, in spite of BP differences. Therefore, the hypothesis to be tested was that testosterone and the hypertensive Yc would increase renal sodium reabsorption.

## Methods

The following 3 studies ask the questions: Study 1-Does testosterone influence urinary sodium and potassium excretion, as well as, systolic BP?; Study 2-Will blocking the androgen receptor mimic the effects of castration on electrolyte excretion?; Study 3-Is there evidence for AR transcripts in the kidney?

### Design

Study 1 utilized a two strain by three treatment design, using SHR/y and WKY males 8 weeks of age. The treatments consisted of gonadally intact controls (control, n = 8/strain), castrates (cast, n = 6/strain), and castrates with testosterone implants (cast+Ti, n = 6/strain). Also 4 age matched females of each strain were tested for 24 hr urinary Na as a comparison to males. Study 2 used two strains-WKY(n = 6) and SHR/y(n = 6), and two treatments-starting with the control period followed by 4 wks of dietary flutamide administration (83 mg/kg body weight; Sigma Chemical Co., St. Louis, MO) in the same animals. Study 3 used kidneys collected from 3 adult males each from two strains (WKY, SHR/y).

### Animal Models

The Y-chromosome (Yc) animal model used in this study consisted of the consomic borderline hypertensive (SHR/y), and normotensive Wistar-Kyoto (WKY) rats [14]. SHR and WKY rats were originally obtained from Harlan Sprague-Dawley (Indianapolis, IN) in 1981 (SHR/hsd and WKY/hsd), and colonies have been maintained at the University of Akron research facility. To develop the SHR/y consomic strain, an SHR male was crossed with a WKY female. A male offspring was then crossed to a WKY female and then a son of this cross was crossed to a WKY female. These crosses have been repeated for over 20 generations, creating the strain designated SHR/y. The repeated crosses have replaced the autosomal and X-linked loci of the parental SHR with the alleles of the maternal WKY strain, while the SHR Y chromosome remains. Therefore, the SHR/y male has an SHR Yc, with the autosomes and an X-chromosome from the WKY female. In comparison, the WKY male has a WKY Yc, with the autosomes and an X-chromosome from the WKY. Comparisons between WKY and SHR/y males allow us to investigate the role of the SHR Yc in a WKY genetic background and any genetic differences between strains must map to the Y chromosomes. For a detailed review, see Ely *et al*. [15]. All animals were treated in a humane manner according to NIH guidelines, and the University of Akron Institutional Animal Care and Use Committee (IACUC) approved all experiments.

### Housing

Animals were housed in pairs according to their strain and maintained in polycarbonate cages (48 cm × 27 cm × 20 cm) with stainless steel covers and heat-treated bedding (R.J. Murphy hardwood Sani Chips). Rats were subjected to a 12 h/12 h light/dark cycle and maintained on a normal sodium diet (0.3% Na, Prolab 3000 Rat/chow 3000, PMI Feeds, St. Louis, MS). Food and water was accessible *ad libitum*. The amount of Na in the food was 0.3% and previous studies have not shown that testosterone treatment increases Na intake.

### Testosterone Experiments

Male rats to be castrated were initially 8 weeks old, sedated with Sodium Brevital (50 mg/kg, IP; E. Lilly, Indianapolis, IN), and both testes were removed. In a similar manner, male rats to be implanted (8 weeks old) were castrated, shaved along the base of the neck, and an incision 0.5 cm long was made. The T implant was inserted beneath the skin parallel to the longitudinal axis of the rat. Briefly testosterone implants [6,7] for castrated rats at 8 weeks of age were prepared by cutting single lumen clear 50 Silastic tubing (0.062" ID × 0.125" OD) into strips that measured 16 mm in length with 12 mm of the tube packed with testosterone propionate (Sigma Chemical Co., St. Louis, MO. Each implant was replaced every two weeks for duration of the experiment [5].

### Testosterone and Aldosterone Assays

Animals were anesthetized at 15 weeks of age with Sodium Brevital (50 mg/kg, IP; E. Lilly, Indianapolis, IN) and a 2–3 ml retro-orbital blood sample was collected between 1100 and 1700 hours and centrifuged for 5 minutes (5,000 ×G) to obtain plasma. Testosterone levels were analyzed by RIA (Bio-Rad Laboratories, Hercules, CA) [4]. The correlation with another kit was r = 0.991, sensitivity was 0.08 ng/mL at the 95% confidence limit, and the highest cross-reactivity with potential interfering steroids was with dihydrotestosterone (6.65%). The coefficient of variation for our sample intra-run was 7.4% to 11.6% and for inter-run was 12.5% to 16.96%.

Aldosterone levels were analyzed in plasma by RIA (Diagnostic Systems Laboratories, Inc. Webster, Texas). Sensitivity was 7.64 pg/mL. The highest cross-reactivity with potential interfering steroids was with corticosterone (0.02%). The coefficient of variation for our sample intra-run was 3.3% to 4.5% and for inter-run was 5.9% to 9.8%.

### Electrolyte Measurement

Plasma was collected for sodium levels in control and castrated animals of each strain at 15 weeks of age using pentothal anesthetic (50 mg/kg, i.p). Urine was collected for 24 hours at 16 weeks of age in order that the volume was not affected by the blood collection at 15 weeks, while the animals were housed in metabolic cages and maintained on their food and water. One ml of mineral oil was placed into each urine-collecting cup prior to the collection period to prevent evaporation of the urine. Sodium and potassium concentrations were measured by flame photometry (Coleman model 51; Bacharach, Inc., Pittsburgh, PA).

### Systolic Blood Pressure

Weekly systolic blood pressure for each strain and treatment group was monitored for the duration of the study. Blood pressure was measured via tail sphygmomanometry and recorded on a physiograph (Desk Model Type DMP 4A, E& M Instruments Co., Inc., Houston, TX) between 1100 and 1400 hrs. The animals were placed into a warming chamber for 20 minutes at 38°C to dilate tail arteries, transferred to a restraint, and a tail cuff was slipped around the base of the tail. Pressures were then determined from the average of 5 systolic blood pressure recordings, which took 2–3 min [3,14].

### Study 3: Is There Evidence for Androgen Receptors in the Kidney?

The objective of this experiment was to determine if androgen receptor (AR) transcripts were present in the kidney of SHR, SHR/y and WKY males. Spontaneously hypertensive rats (SHR) were used to see if WKY and SHR/y rats were similar in AR presence. Gonadally intact males at 15 weeks of age (n = 3/group) from our stock colonies, as previously described, were used to study the presence of AR transcripts in each strain. Animals were anesthetized with pentothal (50 mg/kg, ip) and kidneys removed and placed on foil on dry ice. All animals were housed in a similar manner to study 1.

### Androgen Receptor RT-PCR

Total cellular RNA was isolated from frozen kidneys from SHR, SHR/y and WKY males with RNA STAT-60 (TEL-TEST, Friendswood, TX). For cDNA production the RNA was DNased (Ambion, Austin, TX) with 2 μg used as a template with avian reverse transcriptase (Enhanced Avian HS RT, Sigma Chemical Co., St.Louis, MS), 10× Buffer, dNTPmix, a mix of random nonamers (Sigma Chemical Co., St. Louis, MS), and water to a total volume of 20 μl. RNase inhibitor (SUPER-In, Ambion, Austin, TX) was included to insure RNA integrity. A RT control minus the RT enzyme was included for each sample. The cDNA was amplified at 94°C for 4 min.; 35 cycles of 94°C for 1 min., 52°C for 1 min., 72°C for 1 min.; 72°C for 7 min. Primers were designed from Genbank sequence J05454 for the rat AR receptor gene cDNA. Primer sequences consisted of the reverse primer, DK2 5'TGCTGCCTTCGGATATTACC and the forward primer DK3 5' TAGGGCTGGGAAGGGTCTAC. A positive control (cDNA from testis) and negative control (no template) were included in each set of PCR reactions. PCR products were visually observed on 1% agarose gels and bands of 580 bp were identified as the androgen receptor transcript.

### Statistics

Data were analyzed and graphed with Sigma Statistical Software (Sigma Stat and Sigma Plot, Jandel Scientific Software, San Rafael, CA). Two-way ANOVA using flutamide or T treatment and strain as factors were used to analyze plasma T and aldosterone, urinary electrolytes, and kidney weight related variables with appropriate follow up t-tests (Tukey's or Neuman-Keuls). Significance was assumed with a p < 0.05.

## Results

### Urine Electrolyte Analysis

**Figure 1:**
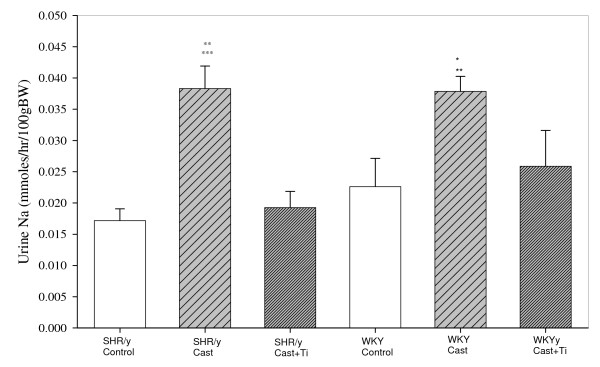
Urinary sodium (Na) concentration (mmol/hr/100 g body weight) for SHR/y and WKY gonadally intact (control, n = 8) males castrate (cast, n = 6), castrate with T implant (cast+Ti, n = 6) at 15 weeks of age, expressed as means, ± SEM. *=p<.05 WKY cast vs. WKY Cast +Ti, **+p<.01 WKY Cast vs. WKY control, **=p<.01 SHR/y cast vs. SHR/y Cast +Ti, ***=p<.001 SHR/y cast vs. SHR/y control.

Figure 1 shows urinary sodium (Na) concentration (mmol/hr/100 g body weight), from study 1, at 15 weeks for each strain and treatment. In both SHR/y and WKY strains, the castrate groups excreted more Na than the control groups and T restored Na excretion toward that of control values (two-way ANOVA: T treatment (F = 10.9, p < 0.001), strain (F = 13.5, p = <0.001), and T treatment x strain interaction (F = 3.3, p = 0.02). There were no significant Na excretion differences between SHR/y and WKY strains for controls or each T treatment. In order to compare for a gender difference in Na excretion, the WKY females excreted about 4× more Na than males: 0.0825 ± 0.014 vs. WKY males 0.024 ± 0.0017 mmol Na/hr/100 gm body weight and similarly SHR/y females excreted about 4X more Na than SHR/y males: 0.0735 ± 0.0088 vs. 0.016 ± 0.001 mmol Na/hr/100 gm body weight (p < 0.05 for both strains).

**Figure 2:**
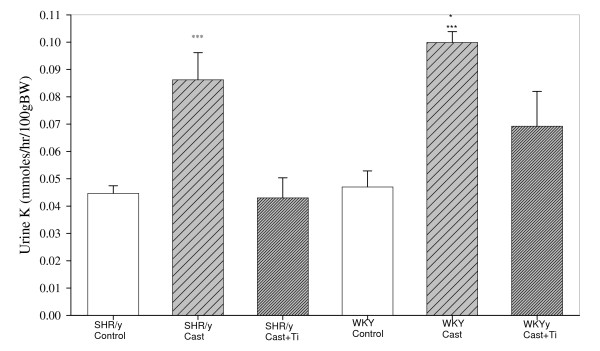
Urinary potassium (K) concentration (mmol/hr/100 g body weight) for SHR/y and WKY gonadally intact control (control, n = 8) males, castrate (cast, n = 6), castrate with T implant (cast+Ti, n = 6) at 15 weeks of age, expressed as means, ± SEM. ***=p<.001 SHR/y cast vs. SHR/control and SHR/y cast+Ti, ***=p<.001 WKY cast vs. WKY control, *=p<.05 WKY cast vs. WKY cast+Ti.

Figure 2 shows urinary potassium (K) concentration (mmol/hr/100 g body weight), from study 1, at 15 weeks for each strain and treatment. In the SHR/y and WKY strains, the castrate groups excreted more K than the control groups and T tended to restore K excretion toward that of the control values (two-way ANOVA: T treatment F = 17.0, p < 0.001, strain F = 17.8, p < 0.001, and T treatment x strain interaction F = 4.9, p = 0.002). There were no significant K excretion strain differences.

### Testosterone, Aldosterone, Blood Pressure and Kidney Weight

Table 1 shows serum testosterone (T) and BP at 15 weeks for each strain and treatment. Testosterone levels were as expected for each treatment with the control groups at 2.5–5.0 ng/ml, the castrate groups at less than 1 ng/ml, and the castrate with T implant groups at 13–15 ng/ml. The two-way ANOVA T results were: T treatment (F = 248.1, p < 0.001), strain (F = 1.4, p = 0.3), and T treatment x strain interaction (F = 2.9, p < 0.05). There was a reduction in BP in both strains after castration and restoration to control values with T addition. The two-way ANOVA BP results were: T treatment (F = 6.2, p = 0.005), strain (F = 8.1, p = 0.001), and T treatment x strain (F = 5.0, p = 0.003). Castration reduced kidney weight and T replacement tended to restore it. The two-way ANOVA for T treatment on relative kidney weight was significant (F = 99.1, p < 0.001).

**Table 1:** Serum Testosterone and Systolic Blood Pressure at 15 Weeks

**Strain**	**Treatment**	**Serum Testosterone (ng/ml)**	**Systolic Blood Pressure (SBP, mmHg)**	**Relative Kidney Weight (mg/100 g BW)**
**SHR/y**	**control**	5.6 +/-1.4	^+++,&&&^155.8+/-3.1	764+/-36.0
**SHR/y**	**cast**	0.05+/-0.02	136.8+/-1.6	^###^616+/-23.0
**SHR/y**	**cast +Ti**	^+++^14.84+/-0.51	142.2+/-2.4	^$$^916+/-19.0
**WKY**	**control**	3.28+/-0.16	137.6+/-2.3	658+/-17.0
**WKY**	**cast**	0.19+/-0.07	^%^126.0+/-4.6	636+/-24.0
**WKY**	**cast +Ti**	^+++^12.44+/-2.00	144.4+/-5.7	783+/-77.0

Serum testosterone (T) levels, systolic blood pressure and relative kidney weight at 15 weeks of age for SHR/y and WKY gonadally intact control (control), castrate (cast) and castrate with T implant (cast+Ti) males, expressed as means +/- SEM. The two-way ANOVA for serum T was significant for T treatment) F = 226.0, p < 0.001). Both T implant groups had elevated T compared to controls (^+++ ^= p < .001). The two-way ANOVA for SBP was significant for T treatment (F = 8.1, p = 0.005), strain (F = 6.2, p = 0.001), and T treatment x strain (F = 4.9, p = 0.003). The two-way ANOVA for T treatment on relative combined kidney weight was significant (F = 99.1, p < 0.001). The SHR/y control group SBP was greater than the SHR/y cast+Ti (^+++ ^= p < 0.01) and SHR/y cast group SBP (^&&&^= p < 0.001). The WKY cast group SBP was less than the WKY cast+Ti and control groups (^% ^= p < 0.05). Relative combined kidney weight for SHR/y cast was significantly less than SHR/y control and SHR/y cast +Ti groups (^### ^= p < 0.001). relative combined kidney weight for the SHR/y cast +Ti group was significantly greater than the SHR/y cast and control groups (^$$ ^= p < 0.01).

**Figure 3:**
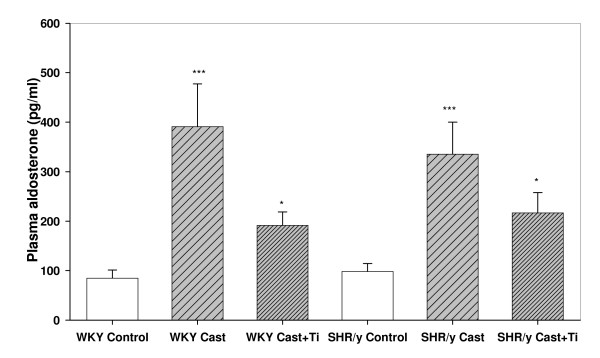
Plasma aldosterone levels (pg/ml) for SHR/y and WKY gonadally intact control (control, n = 8), castrate (cast, n = 6), and castrate with T implant (cast+Ti, n = 6) expressed as means, ± SEM. ***=p<.001 WKY cast vs. WKY control,*=p<.05 WKY cast +Ti vs. WKY cast, *=p<.05 SHR/y cast +Ti vs. SHR/y cast, ***=p<.001 SHR/y cast vs. SHR/y control.

**Figure 4:**
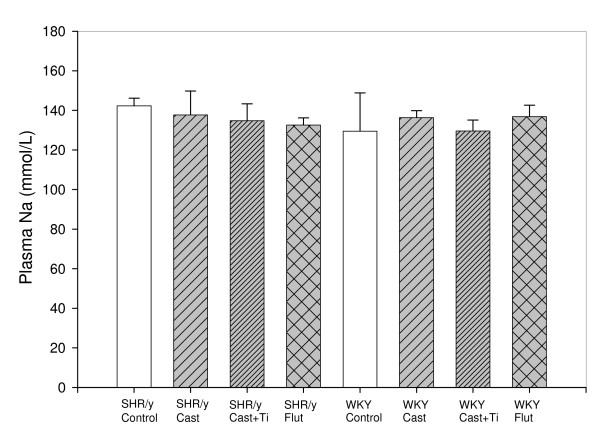
Plasma sodium (Na) levels by treatment group(n = 6/group, means, ± SEM, no significant differences, Two-way ANOVA strain x treatment).

Figure 3 shows plasma aldosterone levels for SHR/y and WKY gonadally intact control, castrate, and castrate with T implant. In both strains castration increased plasma aldosterone and T treatment restored it toward control values. Two-way ANOVA for plasma aldosterone was significant for T treatment (F = 15.6, p < 0.001, t-tests, *p < 0.05 compared to cast, *** = p < .001 compared to control). Figure 4 shows that plasma sodium levels were not significantly altered by strain or treatment effects (2-way ANOVA by strain and treatment).

**Figure 5:**
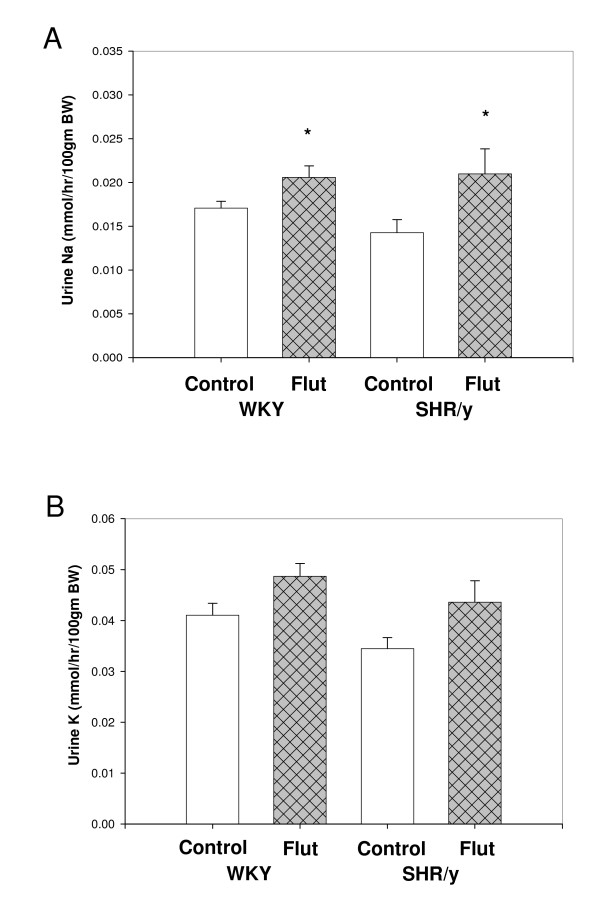
The effect of androgen receptor blockade on urinary NA, K in gonadally intact control (control) WKY and SHR/y males and after flutamide (Flut) treatment (n = 6/group, * = p < 0.05, means, ± SEM).

Figure 5 shows the effect of androgen receptor blockade on urinary Na and K excretion. There was a significant increase in Na excretion in response to androgen receptor blockade in both SHR/y (47%) and WKY (20%) strains. K excretion showed a non significant trend for increase in response to androgen blockade in both strains.

### Androgen Receptor

**Figure 6:**
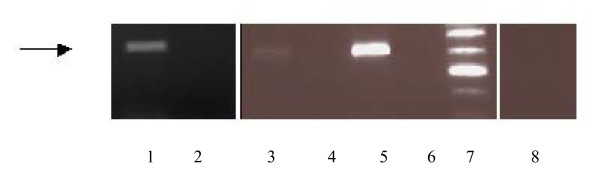
One percent agarose gel of RT-PCR androgen receptor (AR) transcripts taken from SHR (for + control), SHR/y, and WKY kidneys. The arrow indicates the 580bp amplified AR transcript. SHR kidney (lane 1), no RT control (lane 2), WKY kidney (lane 3), no RT control (lane 4), SHR/y kidney (lane 5), no RT control (lane 6), ladder (lane 7), and no template control (lane 8).

The 580 bp amplified AR transcript is present in SHR (+ control), SHR/y and WKY males at 15 weeks of age (Figure 6). Since this method of analysis of RT-PCR is not quantitative, no comparison can be made of levels of expression between strains.

## Discussion

Gender differences in electrolyte excretion have been reported in rats and SHR females excrete more Na than males [16,17]. It makes sense that females would excrete more Na since in most mammals, females have a higher Na intake than males [18]. This may be a protective mechanism to maintain blood volume in times of hemorrhagic injury and during pregnancy, but in resting steady state situations, the Na would need to be excreted. There is evidence that males have more physiological difficulty in responding to blood loss than females [19,20]. Also there are reports of the presence of androgen receptors in female kidneys of rats and mice [21,22]. In our studies, the presence or absence of testosterone (T) altered renal excretion of Na and K in the male borderline hypertensive SHR/y and normotensive WKY strains. In addition, the presence of the androgen receptor (AR) transcripts in the kidney of each strain is consistent with the idea that T binds to the AR in the kidney to play a role controlling Na regulation. With regards to localization of the AR studies have shown AR in proximal and distal tubules [23], and in other locations within the kidney (pars recta and cortical collecting duct (by real-time PCR) and low but detectable levels in medullary and cortical parts of the thick ascending limb of Henle's loop [24].

Androgens could also be operating through the transcriptional control of specific renal genes. Androgen regulated genes in the kidney include several organic anion transporters [23], which could be involved in the androgen produced retention of electrolytes [24,25]. Indeed, Quinkler et al. showed that testosterone increased the expression of the alpha-subunit of the epithelial sodium channel in a human renal proximal tubule cell line 2–3 fold which was blocked by flutamide [26]. This T effect on renal function is not unique to males. Ovarectomized females given T excreted less Na than ovarectomized females [27], further supporting the notion that there may be ARs present in the proximal tubules in both genders [28]. To further support this data we found that SHR females on a high Na diet with T implants excreted less Na than intact females [27]. Plasma Na levels were constant across treatments and cannot be used to explain urinary Na differences. There are many regulatory mechanisms to maintain plasma Na balance so it is not surprising that in spite of Na intake or circulating hormone variations, plasma Na remains constant. For instance, in a study from our lab [5] we found that male, female and testicular feminized males (mutant AR) had the same constant plasma Na levels (145, 146,144 meq/L, respectively) in spite of gender and AR differences.

Androgen receptor blockade produced a significant rise in SHR/y and WKY male Na excretion suggesting a T dependence on Na reabsorption in both strains similar to the effects observed with castration. Flutamide has been used as an anticancer drug and is classified as a nonsteroidal antiandrogen. It competively blocks T at the AR forming inactive complexes which cannot translocate to the nucleus. The increased Na excretion was not as great with flutamide as with castration, which may be due to some ARs still functioning or possibly T working through non-genomic AR mechanisms. Testosterone signaling through non-genomic AR mechanisms has been described in rat osteoblasts [29], AR free macrophages [30], and murine T cells [31,32]; whereby T mediates only ligand-induced Ca^2+ ^import through non-voltage-gated Ca^2+ ^channels [31,32]. Recently, our lab has shown that T via the AR influences Ca^2+ ^signaling through an increase in Ca^2+ ^mobilization in coronary artery adventitial fibroblasts and consequently elevates collagen Type I production [33]. In support of this finding Vicencio [34] has also found that T induces Ca^2+ ^increase by a nongenomic mechanism in cultured rat cardiac myocytes.

Further evidence that T is playing a role in Na reabsorption is provided by the aldosterone data. The removal of T by castration produced a rise in plasma aldosterone and T replacement lowered the aldosterone. This suggests that T was acting to conserve renal Na and its removal caused aldosterone to rise as a compensation. Again, there appears to be a T threshold level for its influence on aldosterone levels since even when T increased 3–4x with replacement the aldosterone levels did not suppress below control values. Indeed, the same results were found in male Sprague Dawley rats castrated and given T replacement [35]. Based on *in vitro *data, T appeared to exert a direct inhibitory effect on basal aldosterone release at the level of adrenal zona glomerulosa cells, but there was no effect on intracellular cAMP [35]. Further studies strongly suggested that the specific inhibitory effect of T on aldosterone was through inhibition of aldosterone synthase [35]. This is further supported by clinical observations in hyperandrogenic women suggesting that circulating T may inhibit adrenal 21- and/or 11B-hydroxylase activity [36]. In our groups that were castrated and received T replacement one might expect to observe greater Na reabsorption since T levels were 3–4x higher with replacement, but the urinary Na levels were comparable to that of the controls for each strain suggesting that there may be a threshold level of T whereby further increases to not affect Na retention mechanisms.

The renin angiotensin system (RAS) and specifically, angiotensinogen (AGT) has been shown to be modulated by sex hormones [37]. Androgens can stimulate synthesis of AGT in animal models [38] and this induction is tissue specific. For instance, androgen has been reported to increase kidney AGT mRNA levels, whereas it has only a slight effect on liver AGT mRNA levels [39]. Androgens may directly upregulate the proximal tubule renin-angiotensin system, increase the volume reabsorptive rate, and thereby increase extracellular volume and blood pressure and secondarily decrease serum angiotensin II levels [40]. We do not have tissue RAS measures, so it is not possible to determine if the T effects are operating through a tissue RAS system in the proximal tubules. Similarily, there is a RAS-dopamine interaction that influences renal proximal tubule cells [41]. Zeng et al. showed that AT1 and D1 receptors interacted differently in renal proximal tubule cells from SHR and WKY [42]. Therefore, it is possible that T can indirectly dopamine to produce renal electrolyte effects. Further research is needed to clarify these RAS-catecholamine interactions.

The same trend of decreased Na excretion in the presence of T was observed with K excretion. K is passively transported across the cell membrane via one of several types of channels in tubules [43]. This was surprising to us since most often there is a reciprocal relationship between renal Na/K exchange. Na and K, are largely exchanged via the Na/K transporter. When the ratio of Na to K excreted was compared, there were no differences between strains or treatment groups. In the SHR/y and WKY strains, the absence of T produced increased K excretion, and T replacement normalized the effect. The AR blockade study showed similar results except the K excretion with the AR blocker was not as great as with castration. Most likely this was due to not all of the ARs are functionally blocked with the drug dosage used. Also relative kidney weight cannot explain the excretion differences found with testosterone manipulation, since the WKY strain did not have significant kidney weight changes but did have excretion differences. The decrease in kidney weight in the SHR/y strain that was castrated and its rescue in the castrates treated with testosterone supports the findings of others who have shown an anabolic effect of testosterone on kidney weight [44-46].

Blood pressure cannot be used to explain the electrolyte excretion differences with T manipulation since BP decreased after castration and Na excretion increased. The BP profile for each strain and treatment group is similar to previously reported data from our lab [4,5,47] showing decreased BP with castration and T treatment restored BP to control or above control levels. In a Na balance study comparing SHR and WKY males, Lundin *et al*. [12] did not find increased SHR urinary Na excretion as expected due to high BP, but instead a decreased urinary Na excretion was found. Similar SHR urinary Na excretion results were found by Osborn *et al*. [13] with a reduced renal ability to excrete Na, in spite of increased blood pressure. Since T was not measured in Osborn's studies, it is not known if the reduced Na excretion may have been due to elevated T.

## Conclusion

The excretion of Na and K in both SHR/y and WKY males appear to be partially T dependent. In addition, the elevated Na excretion in SHR/y and WKY following castration, further suggests that T can be an important player in renal Na excretion. These differences in renal excretion of Na and K cannot be solely and consistently explained by changes in BP or anabolic effects on kidney weight. In conclusion, the increased Na and K excretion following castration, identification of renal AR transcripts, and loss of Na with androgen blockade suggests that T is involved in the regulation of Na balance.

## Competing interests

The author(s) declare that they have no competing interests.

## Authors' contributions

DE, AM, JT, CJ and MT designed and directed studies, were involved in the analysis of the data, and writing of the manuscript. GD, JT, MH and SB performed the experiments and ran the assays. All authors have read and approved the manuscript.

## References

[B1] Lemne CE (1998). Increased blood pressure reactivity in children of borderline hypertensive fathers. J Hypertens.

[B2] Uehara Y, Shin WS, Watanabe T, Osanai T, Miyazaki M, Kansae H, Taguchi R, Sogano K, Toyo-Oka T (1998). A hypertensive father, but not a hypertensive mother, determines blood pressure in normotensive offspring through body mass index. J Hum Hypertens.

[B3] Ely DL, Turner ME (1990). Hypertension in the spontaneously hypertensive rat is linked to the Y-chromosome. Hypertension.

[B4] Ely DL, Falvo J, Dunphy G, Caplea A, Salisbury R, Turner M (1994). The spontaneously hypertensive rat Y-chromosome produces an early testosterone rise in normotensive rats. J Hypertens.

[B5] Ely DL, Salisbury R, Hadi D, Turner M, Johnson M (1991). Androgen receptor and the testes influence hypertension in a rat hybrid model. Hypertension.

[B6] Ely DL, Caplea A, Dunphy G, Daneshavar H, Turner M, Milsted A, Takiyyuddin M (1997). Spontaneously hypertensive rat Y-chromosome increases indexes of sympathetic nervous system activity. Hypertension.

[B7] Jones TJ, Dunphy G, Milsted A, Ely DL (1998). Testosterone effects on renal norepinephrine content and release. Hypertension.

[B8] Reckelhoff JF, Zhang H, Srivastava K, Granger JP (1999). Gender differences in hypertension in spontaneously hypertensive rats: role of androgens and androgen receptor. Hypertension.

[B9] Granger J (1999). Regulation of extracellular fluid volume by integrated control of sodium excretion. Adv Physiol Educ.

[B10] Cowley AW, Roman RJ (1996). The role of kidney in hypertension. JAMA.

[B11] Lumbers ER (1999). Angiotensin and aldosterone. Regul Pept.

[B12] Lundin S, Jerlitz H, Hallback-Nordlander M, Ricksten S, Gothberg G, Berglund G (1982). Sodium balance during development of hypertension in the spontaneously hypertensive rat (SHR). Acta Physiol Scand.

[B13] Osborn JL (1991). Relation between sodium intake, renal function, and the regulation of arterial pressure. Hypertension.

[B14] Ely DL, Daneshvar H, Turner ME, Johnson M, Salisbury R (1993). The hypertensive Y-chromosome elevates blood pressure in the F_11 _normotensive rat. Hypertension.

[B15] Ely DL, Turner ME, Milsted A (2000). Review of the Y-chromosome and hypertension. Braz J Med and Biol Res.

[B16] Chen Z, Vaughn DA, Fanestil DD (1994). Influence of gender on renal thiazide diuretic receptor density and response. J Am Soc Nephro.

[B17] Reckelhoff JF, Zhang H, Granger JP (1998). Testosterone exacerbates hypertension and reduces pressure-natriuresis in male spontaneously hypertensive rats. Hypertension.

[B18] Kensicki E, Dunphy G, Milsted A, Ely D (2002). Estradiol increases sodium intake in female normotensive and hypertensive rats. J Appl Physiol.

[B19] Knoferl M, Angele M, Diodato M, Schwacha M, Ayala A, Cioffi W, Bland K, Chaudry I (2002). Female Sex Hormones Regulate Macrophage Function After Trauma-Hemorrhage and Prevent Increased Death Rate From Subsequent Sepsis. Surgical Technique. Annals of Surgery.

[B20] Angele MK, Schwacha MG, Ayala A, Chaudry IH (2000). Effect of gender and sex hormones on immune responses following shock. Shock.

[B21] Nagao S, Kusaka M, Nishii K, Marunouchi T, Kurahashi H, Takahashi H, Grantham J (2005). Androgen receptor pathway in rats with autosomal dominant polycystic kidney disease. J Am Soc Nephrol.

[B22] Pajunen AE, Isomaa VV, Janne OA, Bardin CW (1982). Androgenic regulation of ornithine decarboxylase activity in mouse kidney and its relationship to changes in cytosol and nuclear androgen receptor concentrations. J Biol Chem.

[B23] Takeda H, Mizuno T, Lasnitzki I (1985). Autoradiographic. studies of androgen-binding sites in the rat urogenital sinus and postnatal prostate. J Endocrinol.

[B24] Le S, Moellic C, Blot-Chabaud M, Farman N, Courtois-Coutry N (2005). Expression of androgen receptor and androgen regulation of NDRG2 in the rat renal collecting duct. Pflugers Arch – Eur J Physiol.

[B25] Kontula KK, Seppanen PJ, Van Duyne P, Bardin CW, Janne OA (1984). Effect of a nonsteroidal antiandrogen, Flutamide, on androgen receptor dynamics and ornithine decarboxylase gene expression mouse kidney. Endocrinology.

[B26] Crocoll A, Zhu CC, Cato AC, Blum M (1998). Expression of androgen receptor mRNA during mouse embryogenesis. Mech Dev.

[B27] Wilson CM, McPhaul MJ (1996). A and B forms of the androgen receptor are expressed in a variety of human tissues. Mol Cell Endocrinol.

[B28] Quinkler M, Buljalska IJ, Kaur K, Onyimba CV, Buhner S, Allolio B, Hughes SV, Hewison M, Stewart PM (2005). Androgen receptor-mediated regulation of the alpha-subunit of the epithelial sodium channel in human kidney. Hypetens.

[B29] Liu B (1997). The effects of testosterone and estrogen on the development of hypertension in female SHR on a high sodium diet.

[B30] Reckelhoff JF, Granger JP (1999). Role of androgens in mediating hypertension and renal injury. Clin Exp Pharmacol Physiol.

[B31] Lieberherr M, Grosse B (1994). Androgens increase intracellular calcium concentration and inositol 1,4,5-trisphosphate and diacylglycerol formation via a pertussis toxin-sensitive G-protein. J Biol Chem.

[B32] Benten WPM, Lieberherr M, Stamm O, Wrehlke C, Guo C, Wunderlich F (1999). Testosterone Signaling through Internalizable Surface Receptors in Androgen Receptor-free Macrophages. MBC Online.

[B33] Benten WPM, Lieberherr M, Sekeris CE, Wunderlich F (1997). Testosterone induced Ca2+ influx via nongenomic surface receptors in activated T cells. FEBS Lett.

[B34] Benten WPM, Lieberherr M, Giese G, Wrehlke C, Stamm O, Sekeris CE, Mossmann H, Wunderlich F (1999). Functional testosterone receptors in plasma membranes of T cells. FASEB J.

[B35] Jenkins C, Milsted A, Doane K, Meszaros G, Toot J, Ely D (2007). A cell culture model using rat coronary artery adventitial fibroblasts to measure collagen production. BMC Cardiovascular Disorders.

[B36] Vicencio JM, Ibarra C, Estrada M, Chiong M, Soto D, Parra V, Diaz-Araya, Jaimovich E, Lavandero S (2006). Testosterone Induces an Intracellular Calcium Increase by a Nongenomic Mechanism in Cultured Rat Cardiac Myocytes. Endocrinol.

[B37] Kau M-K, Lo M-J, Wang S-W, Tsai S-C, Chen J-J, Chiao Y-C, Yeh J-Y, Lin H, Shum AY-C, Fang VS, Ho L-T, Wang PS (1999). Inhibition of Aldosterone Production by Testosterone in Male Rats. Metabolism.

[B38] Givens JR, Andersen RN, Ragland JB, Wiser WL, Umstot ES (1975). Adrenal function in hirsutism. I. Diurnal change and response of plasma androstenedione, testosterone,17-hydroxyprogesterone, cortisol, LH and FSH to dexamethasone and 1/2 unit of ACTH. J Clin Endocrinol Metab.

[B39] Lynch KR, Peach MJ (1991). Molecular biology of angiotensinogen. Hypertension.

[B40] Klett C, Hellmann W, Hackenthal E, Ganten D (1993). Modulation of tissue angiotensinogen gene expression by glucocorticoids, estrogens, and androgens in SHR and WKY rats. Clin Exp Hypertens.

[B41] Ellison KE, Ingelfinger JR, Pivor M, Dzau VJ (1989). Androgen regulation of rat renal angiotensinogen messenger RNA expression. J Clin Invest.

[B42] Zeng C, Wang Z, Hopfler U, Asico LD, Eisner GM, Felder RA, Jose PA (2005). Rat strain effects of AT_1 _receptor activity on D_1 _dopamine receptor in immortalized renal proximal tubule cells. Hyper.

[B43] Quan A, Chakravarty S, Chen J-K, Chen J-C, Loleh S, Saini N, Harris RC, Capdevila J, Quigley R (2004). Androgens augment proximal tubule Transport. Am J Physiol Renal Physiol.

[B44] Tovar A, Sanchez-Capelo A, Cremades A, Peñafiel R (1995). An evaluation of the role of polyamines in different models of kidney hypertrophy in mice. Kidney Int.

[B45] Fine LG, Norman J (1989). Cellular events in renal hypertrophy. Annual Review of Physiology.

[B46] Manteuffel-Cymorowska M, Grzelakowska W, Sztabert B (1993). Polyamines in testosterone-induced hypertrophic and antifolate-induced hyperplastic mouse kidney. Biochim Biophys Acta.

[B47] Jenkins C, Salisbury R, Ely D (1994). Castration lowers and testosterone restores blood pressure in several rat strains on high sodium diets. Clin Exper Hyper.

